# Longitudinal faster anxiety progression of *GBA* variant carriers in the early Parkinson’s disease cohort

**DOI:** 10.3389/fnins.2024.1353759

**Published:** 2024-01-24

**Authors:** Shushan Sang, Yunpeng Ba, Nannan Yang

**Affiliations:** ^1^Department of Otolaryngology-Head and Neck Surgery, The First Affiliated Hospital of Zhengzhou University, Zhengzhou, China; ^2^Department of Neurology, The First Affiliated Hospital of Zhengzhou University, Zhengzhou, China

**Keywords:** Parkinson’s disease, anxiety, GBA variants, dysautonomia, pathogenic mechanism

## Abstract

**Objective:**

Anxiety symptoms are prevalent neuropsychiatric manifestations in Parkinson’s disease (PD) and impact the development of motor complications. Our aim was to evaluate the association of *GBA* variants with the anxiety development in early PD cohort.

**Methods:**

This cohort study used data from the Parkinson Progression Marker Initiative. The primary outcome anxiety was assessed by State–Trait Anxiety Inventory (STAI). The association between *GBA* and longitudinal change in the STAI total score was examined using linear mixed-effects model, and the association between *GBA* and anxiety progression was examined using Cox survival analysis.

**Results:**

A total of 385 patients with PD were included in this study, 39 of them were *GBA* variant carriers and 346 were idiopathic PD without *GBA* variants. Patients with *GBA* variants had faster annual increase in anxiety score (*β* = 0.44; 95% CI, 0.18 to 0.71; *p* < 0.001) and were at higher risk of anxiety progression (HR 1.87; 95% CI, 1.03 to 3.41; *p* = 0.03,). Higher baseline scores for Scales for Outcomes in Parkinson’s Disease-Autonomic (SCOPA-AUT), which indicated the autonomic dysfunction, also independently predicted faster increase in anxiety score (*β* = 0.48; 95%CI, 0.19 to 0.69; *p* < 0.001) and higher incidence of anxiety development (HR = 1.05; 95% CI, 1.01 to 1.08; *p* = 0.008).

**Interpretation:**

These findings suggest that longitudinal anxiety symptoms worsening was faster in PD patients who were *GBA* variant carriers and have dysautonomia, and this association was enhanced if they have both.

## Introduction

Anxiety represents a highly prevalent and subjectively burdensome non-motor symptom in Parkinson’s disease (PD) (Blundell et al., 2023), with an average prevalence of 31% and a lifetime prevalence reaching up to 49% (Broen et al., 2016; Fan et al., 2022). Anxiety often emerges prior to the onset of motor symptoms in PD, suggesting its potential as an early manifestation of the disease (McGregor and Nelson, 2019; Carey et al., 2021). Anxiety in PD patients not only affects emotional wellbeing and daily functioning but also predict the severity of motor complications and cognitive decline, which further decreased health-related quality of life and increased more caregiver burden (Hinkle et al., 2021; Lo Buono et al., 2021).

Although the exact pathological mechanism of anxiety in Parkinson’s disease (PD) is not fully understood, researchers have proposed various predictors of anxiety in PD (Leentjens et al., 2011; Khedr et al., 2020). However, it is worth noting that most studies investigating this subject have been cross-sectional in nature (Dissanayaka et al., 2010; Cui et al., 2017). Only a few studies have incorporated analyses of longitudinal data to ascertain predictors of anxiety in PD, including baseline probable REM sleep behavior disorder (pRBD), cognitive impairment, daytime sleepiness, dysautonomia, and depressive symptoms (Rutten et al., 2017; Zhu et al., 2017; Wang et al., 2023), but the influence of genetic variants on anxiety development in PD patients over time has been poorly studied yet.

The *GBA* gene is responsible for encoding the lysosomal enzyme, β-glucocerebrosidase (GCase), which plays a crucial role in the metabolism of sphingolipids (Behl et al., 2021; Vázquez-Vélez and Zoghbi, 2021). *GBA* variants have emerged as the predominant genetic risk factors for PD on a global scale, and PD patients carrying *GBA* variants generally exhibit earlier onset, more severe motor and gait impairments, an increased risk of cognitive decline and depression, faster disease progression, and reduced survival rates (Neumann et al., 2009; Brockmann et al., 2015; Petrucci et al., 2020; Yang et al., 2023). However, the relationship between the *GBA* variants and anxiety progression remains unclear. Therefore, the aim of this study was to examine the associations between *GBA* variants, clinical covariates, and the anxiety progression in a large group of patients with early PD.

## Methods

### Study population

This study obtained data from the Parkinson’s Progression Markers Initiative (PPMI) database, and the PPMI study is an ongoing observational longitudinal study which aims to identify progression biomarkers for PD (Marek et al., 2018). Study protocols and manuals are available online at.^1^ Patients were recruited between 2010 and 2018 based on the strict inclusion/exclusion criteria that were previously described (Marek et al., 2018; Yang et al., 2022a). We downloaded data from the PPMI database in December 2022, and 5 years of follow-up data were included in further analysis.

A total of 385 idiopathic PD patients who had follow-up data were involved in the linear mixed model analysis, including 39 PD patients with *GBA* variants (GBA-PD) and 346 idiopathic PD without *GBA* variants (iPD). The GBA-PD group included those most common variants: N370S, L444P, T369M, and E326K (Straniero et al., 2020; Hoglinger et al., 2022; Pang et al., 2022).

### Genetic testing

DNA was extracted from the whole blood of the patients according to the study protocol as described in the PPMI biologics manual.^2^ Sanger sequencing was performed by PPMI to analyze and screen for variants within exons 1–11 of the *GBA* gene.

### Assessment of clinical covariates

We obtained demographic and clinical data from the PPMI database for all subjects. Clinical evaluation was performed in the “off” treatment state. Motor symptoms were evaluated with the MDS-UPDRS III (Goetz et al., 2007). Global cognitive status was assessed using the Montreal Cognitive Assessment (MoCA) (Nasreddine et al., 2005). Autonomic dysfunction was assessed with the Scales for Outcomes in Parkinson’s Disease-Autonomic (SCOPA-AUT) (Rodriguez-Blazquez et al., 2010). Olfactory dysfunction was measured by the University of Pennsylvania Smell Identification Test (UPSIT) (Vengalil et al., 2016). Depressive symptoms were assessed with the short version of the Geriatric Depression Scale (GDS), and probable depression was defined as GDS >5 (Paudel et al., 2017). Excessive daytime sleepiness (EDS) was assessed with the Epworth Sleepiness Scale (ESS), and ESS > 10 was defined as EDS (Büchele et al., 2018). REM Sleep Behavior Disorder (RBD) was assessed by REM Sleep Behavior Disorder Screening Questionnaire (RBDSQ), and RBDSQ >5 was defined as probable REM Sleep Behavior Disorder (pRBD) (Berg et al., 2021).

The main outcome anxiety was assessed with State–Trait Anxiety Inventory (STAI). It consists of two subscales, the State subscale and the Trait subscale, with a total score for each subscale ranging from 20 to 80. The State subscale measures the current state of anxiety, whereas the Trait subscale evaluates relatively stable aspects of “anxiety proneness.” The STAI total score is a sum of the State subscale and the Trait subscale (Julian, 2011). It has been reported that the STAI had a good internal consistency among *de novo* PD patients (Yang et al., 2019), and clinically relevant anxiety is commonly defined as a STAI State score of ≥39 (Knight et al., 1983).

### Statistical analysis

The association between *GBA* variants and longitudinal changes in STAI total scores was assessed using multivariate linear mixed-effects models in R 4.2.1 with the lme4 package. Development of anxiety was evaluated using Cox regression (package survival) and visualized in Kaplan–Meier plots (package survminer) in R 4.2.1. Patients were censored due to death, loss to follow-up, or last recorded visit. Models were adjusted for confounders, such as age at onset, sex, and education. All baseline variables with value of ps of less than 0.1 in the univariable Cox models were included in subsequent multivariable Cox models with a backward elimination procedure. The cutoff value of continuous variable such as baseline SCOPA-AUT scores that producing the maximum log rank was defined using the R package “maxstat.” Differences in demographic characteristics between the GBA-PD group and the iPD group were assessed using Student’s *t*-test or the Mann–Whitney *U* test for continuous variables and the *χ*2 test for categorical variables in IBM SPSS 25.0 (Armonk, NY, USA). All *p*-values were two-sided, and a *p*-value of less than 0.05 was regarded as statistically significant.

## Results

### Study population

The demographic and clinical characteristics of the participants included in this study are presented in Table 1, including 39 PD patients with *GBA* variants (GBA-PD group) and 346 idiopathic PD without *GBA* variants (iPD group). At the baseline, the GBA-PD group showed higher RBDSQ scores (*p* = 0.006).

**Table 1:** Characteristics of the *GBA* variants carriers and iPD

Characteristics variables	Mean (*SD*)		*p* value
	iPD (*n* = 346)	GBA-PD (*n* = 39)	
Age, y	61.8 (9.6)	58.9 (9.5)	0.07
Male, *n* (%)	110 (31.8)	16 (41.0)	0.24
Disease duration, y	0.56 (0.54)	0.52 (0.55)	0.30
Age at onset	59.8 (9.7)	56.9 (9.9)	0.07
STAI baseline	56.2 (10.4)	58.5 (10.7)	0.28
MDS-UPDRS III	20.4 (8.7)	23.8 (11.3)	0.16
PIGD subtype, *n* (%)	101 (29.2)	11 (28.2)	0.89
SCOPA-AUT	9.7 (6.8)	10.3 (7.1)	0.71
UPSIT score	22.4 (8.2)	21.7 (8.0)	0.49
MoCA score	27.0 (2.4)	26.7 (2.4)	0.25
RBDSQ score	3.9 (2.6)	5.8 (3.0)	**< 0.001**
pRBD (RBDSQ>5)	84 (24.2)	18 (46.2)	**0.003**
GDS score	2.4 (2.6)	2.7 (2.8)	0.57
Depression (GDS>5)	40 (11.6)	6 (15.3)	0.66
ESS score	5.9 (3.5)	6.1 (3.7)	0.76
EDS (ESS>10)	55 (15.9)	8 (20.5)	0.46
Caudata DAT	2.0 (0.58)	1.9 (0.64)	0.14
Putamen DAT	0.88 (0.41)	0.76 (0.30)	0.06
CSF Aβ_42_	889.3 (421.8)	807.9 (341.6)	0.48
CSF a-syn	1473.1 (691.1)	1315.2 (621.4)	0.25
CSF t-tau	161.1 (66.8)	144.4 (65.2)	0.32
CSF p-tau	13.3 (5.8)	12.0 (5.9)	0.39

Data are shown as mean ± standard deviation or prevalence (%); Abbreviations: GBA, β-glucocerebrosidase; STAI, State-Trait Anxiety Inventory; MDS-UPDRS III, Movement Disorders Society Unified Parkinson’s Disease Rating Scale part3; PIGD, postural instability gait difficulty; SCOPA, Scale for Outcomes in Parkinson disease Autonomic; RBDSQ, REM Sleep Behavior Disorder Screening Questionnaire; UPSTI, University of Pennsylvania Smell Identification Test; GDS, Geriatric Depression Scale; MoCA, Montreal Cognitive Assessment; ESS, Epworth sleepiness Scale; EDS, Excessive Daytime Sleepiness; DAT, dopamine transporter; CSF, cerebrospinal fluid; Aβ42, β-amyloid 1-42; α-syn, total alpha-synuclein; t-tau, total tau; p-tau, phosphorylated tau. Values in bold indicate statistically significant results.

### Faster anxiety-STAI total score increase in *GBA* variant carriers

**Figure 1:**
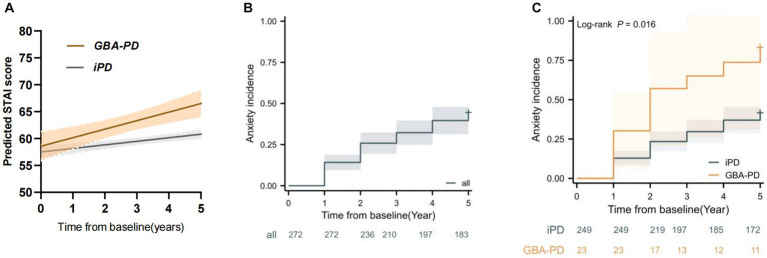
*GBA* variant carriers showed a faster increase in anxiety score and higher risk of anxiety progression. **(A)** Longitudinal trajectories of mean state–trait anxiety inventory (STAI) ratings in *GBA* variant carrier and non-carrier groups **(B)** Kaplan–Meier analyses of anxiety progression in Parkinson’s disease patients. **(C)** Kaplan–Meier analyses of anxiety progression in *GBA* variant carrier and non-carrier groups.

Linear mixed models demonstrated a more rapid Anxiety-STAI total score increase than non-carriers, with an annual increase in STAI total score of 1.21 point (95% CI, 0.41 to 2.1 point) versus 0.77 point (95% CI, 0.26 to 0.99 point), respectively. This difference was significant (*β* = 0.44; 95% CI, 0.18 to 0.71; *p* < 0.001) (Table 2, Figure 1A). A similar relationship was observed in the adjusted model 2 (including demographic factors, medication, cognition, motor symptoms, autonomic symptoms, sleep disorder, olfactory symptoms, and depression factor). Baseline SCOPA-AUT total score and depression and postural instability gait difficulty (PIGD) subtype were also significantly associated with faster increase in annual anxiety score in this linear mixed-effects model (Table 2). One point increase in SCOPA-AUT score was associated with an increase of 0.48 point in the STAI total score (*p* = 0.001). Baseline UPSIT score, MoCA score, and age at onset were also significantly independent predictors of annual change in STAI total score. One unit increase in age at onset, UPSIT score, and MoCA score was associated with a decrease of 0.22, 0.25, and 0.71 point in the anxiety score (*p* = 0.002, *p* = 0.02, and *p* = 0.03, respectively) (Table 2).

**Table 2:** Linear mixed model for predicting annual change in State–Trait Anxiety Inventory (STAI) score.

Model	β-coefficient (95% CI)	*p*-value
Model 1^a^		
*GBA* variants		
Main effect	−0.21 (−1.0 ~ 0.99)	0.92
Interaction with time	0.44 (0.18 to 0.71)	**<0.001**
Model 2^b^		
*GBA* interaction with time	0.24 (0.04 to 0.43)	**<0.001**
Male	2.7 (−0.28 to 5.8)	0.08
Baseline MDS-UPDRS III	0.02 (−0.14 to 0.19)	0.8
LEDD over time	−0.000 (−0.002 to 0.0009)	0.31
Age at onset	−0.25 (−0.42 to-0.09)	**0.002**
PD subtype (PIGD)	3.30 (0.20 to 6.40)	**0.04**
Baseline SCOPA-AUT	0.48 (0.19 to 0.69)	**0.001**
Baseline UPSIT score	−0.22 (−0.41 to-0.10)	**0.02**
Baseline MoCA score	−0.71 (−1.34 to-0.08)	**0.03**
Baseline Depression	23.2 (18.3 to 28.1)	**<0.001**
Baseline RBD	3.0 (−0.35 to 6.37)	0.09
Baseline EDS	1.8 (−2.2 to 5.9)	0.32
Baseline Caudate DAT	−1.8 (−5.7 to 2.0)	0.34
Baseline Putamen DAT	4.6 (−3.2 to 12.5)	0.31
Baseline CSF tau	0.06 (−0.005 to 0.14)	0.09
Baseline CSF α-syn	−0.001 (−0.005 to 0.002)	0.41
Baseline CSF Aβ_42_	−0.002 (−0.006 to 0.002)	0.43
Baseline CSF p-tau	−0.51 (−1.2 to 0.16)	0.16

CI, confidence interval; GBA, β-glucocerebrosidase; MDS-UPDRS III, Movement Disorders Society Unified Parkinson’s Disease Rating Scale part3; LEDD, Total Levodopa Equivalent Daily Dose; PIGD, postural instability gait difficulty; SCOPA, Scale for Outcomes in Parkinson disease Autonomic; RBDSQ, REM Sleep Behavior Disorder Screening Questionnaire; UPSTI, University of Pennsylvania Smell Identification Test; GDS, Geriatric Depression Scale; MoCA, Montreal Cognitive Assessment; ESS, Epworth sleepiness Scale; EDS, Excessive Daytime Sleepiness; DAT, dopamine transporter; CSF, cerebrospinal fluid; Aβ42, β-amyloid 1–42; α-syn, total alpha-synuclein, t-tau, total tau; p-tau, phosphorylated tau. Values in bold indicate statistically significant results. ^a^means this model GBA and GBA interaction with time as random factor and age at onset, male as fixed factor; ^b^means this model GBA interaction with time as random factor, LEDD over time, and other clinical covariate were all baseline data.

To further find the association between the clinical covariate changes over time and changes in anxiety scores during time, another linear mixed model was performed including those clinical covariate scores over time, and the cerebrospinal fluid total-tau (CSF t-tau), LEDD over time, and MDS-UPDRS motor score were associated with anxiety score (Table 3).

**Table 3:** Linear mixed model for predicting annual change in State–Trait Anxiety Inventory (STAI) score with clinical covariates change over time.

Model	β-coefficient (95% CI)	*p*-value
*GBA* interaction with time	0.40 (0.19 to 0.57)	**<0.001**
LEDD over time	−0.003 (−0.006 to-0.0009)	**0.009**
Age at onset	−0.14 (−0.29 to 0.0003)	0.051
PD subtype (PIGD)	2.9 (−0.23 to 6.4)	0.07
MDS-UPDRS III over time	0.06 (0.003 to 0.11)	**0.039**
SCOPA-AUT over time	0.50 (0.34 to 0.65)	**<0.001**
MoCA over time	−0.27 (−0.53 to-0.01)	**0.042**
Baseline Depression	24.0 (19.3 to 29.0)	**<0.001**
Baseline RBD	3.4 (0.11 to 6.81)	**0.043**
Baseline EDS	1.9 (−2.1 to 6.0)	0.35
CSF t-tau over time	−0.023 (−0.045 to-0.002)	**0.028**
CSF α-syn over time	0.004 (−0.001 to 0.002)	0.64
CSF Aβ_42_ over time	0.001 (−0.001 to 0.004)	0.37

CI, confidence interval; GBA, β-glucocerebrosidase; MDS-UPDRS III, Movement Disorders Society Unified Parkinson’s Disease Rating Scale part3; LEDD, Total Levodopa Equivalent Daily Dose; PIGD, postural instability gait difficulty; SCOPA, Scale for Outcomes in Parkinson disease Autonomic; RBDSQ, REM Sleep Behavior Disorder Screening Questionnaire; UPSTI, University of Pennsylvania Smell Identification Test; GDS, Geriatric Depression Scale; MoCA, Montreal Cognitive Assessment; EDS, Excessive Daytime Sleepiness; CSF, cerebrospinal fluid; Aβ42, β-amyloid 1–42; α-syn, total alpha-synuclein, t-tau, total tau. Values in bold indicate statistically significant results.

### *GBA* affect the anxiety progression

As we mentioned in the method part, clinically relevant anxiety is commonly defined as a STAI State score of ≥39; 97 patients from the iPD group and 16 from the GBA-PD group were found to have anxiety at baseline and were excluded from further survival analysis. Finally, 272 participants were enrolled in the survival analysis, including 23 patients from the GBA-PD group and 249 patients from the iPD group. The baseline characteristics of those 272 patients are presented in Supplementary Table S1.

During the 5-year follow-up, 98 patients (36.02%) reported developing anxiety (Figure 1B), 13 patients were from the GBA-PD group and 85 patients from the iPD group. Kaplan–Meier estimates showed that the GBA-PD group had a significantly higher incidence of anxiety than the iPD group (log rank 5.844, *p* = 0.016) (Figure 1C).

The results of the Cox regression analyses are shown in Table 4. In the univariable Cox regression analyses, the *GBA* variants, dysautonomia, pRBD, cognitive impairment, and olfactory dysfunction were significantly associated with anxiety progression. In multivariable Cox model, *GBA* variants remained to be a significant predictor of anxiety progression (HR 1.87; 95% CI 1.03 to 3.41; *p* = 0.03, Table 4) and dysautonomia (HR = 1.05; 95% CI 1.01 to 1.08; *p* = 0.008, Table 4), independently increasing the risk of anxiety development.

**Table 4:** Results of COX regression analyses for the predictors of anxiety progression.

Variables	Univariable analysis		Multivariable analysis	
	HR (95% CI)	*p* value	HR (95% CI)	*p*-value
GBA-PD	2.06 (1.15 to 3.70)	**0.01**	1.87 (1.03 to 3.41)	**0.03**
Male	1.09 (0.715 to 1.67)	0.67	—	—
Age at onset	0.99 (0.97 to 1.02)	0.60	—	—
MDS-UPDRS III	0.99 (0.97 to 1.02)	0.54	—	—
PD subtype (TD)	0.75 (0.48 to 1.16)	0.20	—	—
SCOPA-AUT score	1.06 (1.03 to 1.09)	**<0.001**	1.05 (1.01 to 1.08)	**0.008**
UPSIT score	0.97 (0.95 to 1.002)	**0.05**	0.98 (0.95 to 1.003)	0.11
MOCA score	0.91 (0.83 to 0.98)	**0.02**	0.93 (0.85 to 1.01)	0.10
pRBD	1.88 (1.23 to 2.87)	**0.003**	1.36 (0.85 to 2.19)	0.19
Depression	2.18 (0.95 to 4.98)	0.06	1.76 (0.74 to 4.18)	0.20
EDS	1.34 (0.79 to 2.26)	0.27	—	—
Caudate DAT	0.91 (0.64 to 1.30)	0.62	—	—
Putamen DAT	0.67 (0.36 to 1.25)	0.21	—	—
CSF α-syn	1.00 (1.00 to 1.00)	0.99	—	—
CSF Aβ_42_	1.00 (0.99 to 1.00)	0.28	—	—
CSF t-tau	1.00 (0.99 to 1.00)	0.55	—	—
CSF p-tau	1.00 (0.97 to 1.03)	0.98	—	—

Only baseline variables with p value less than 0.1 in the univariable Cox models were included in subsequent multivariable Cox models. CI, confidence interval; GBA, β-glucocerebrosidase; MDS-UPDRS III, Movement Disorders Society Unified Parkinson’s Disease Rating Scale part3;TD subtype, tremor dominant type; SCOPA-AUT, Scale for Outcomes in Parkinson disease Autonomic; UPSIT score, University of Pennsylvania Smell Identification Test; Depression, GDS > 5; MCI, MOCA (17, 26); pRBD, probable REM Sleep Behavior Disorder, REM Sleep Behavior Disorder Screening Questionnaire > 5; EDS, excessive daytime sleepiness, Epworth sleepiness Scale;>9; DAT, The dopamine transporter; CSF, cerebrospinal fluid; α-syn, total alpha-synuclein; Aβ_42_, β-amyloid 1–42; t-tau, total tau; p-tau, phosphorylated tau. Values in bold indicate statistically significant results.

### Dysautonomia affect the anxiety progression

**Figure 2:**
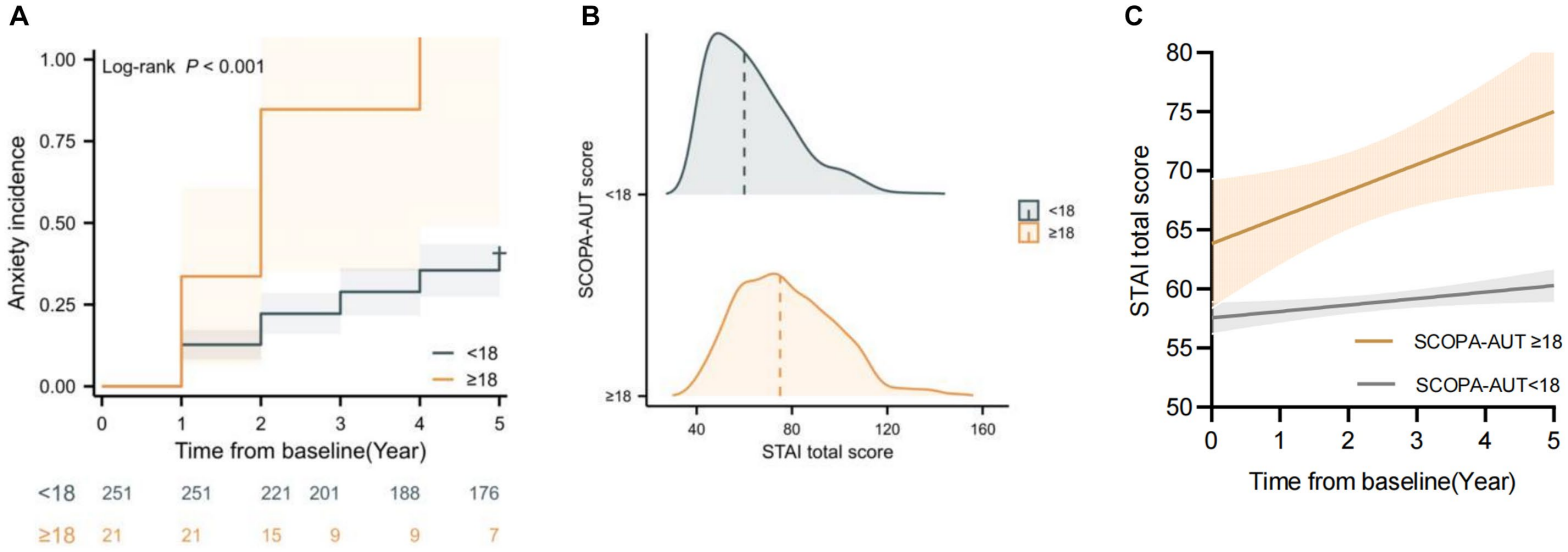
Baseline Scales for Outcomes in Parkinson’s Disease-Autonomic (SCOPA-AUT) was associated with State–Trait Anxiety Inventory (STAI) change and anxiety progression over time. **(A)** Kaplan–Meier analyses of anxiety progression in different SCOPA-AUT level groups. **(B)** Raincloud plots showing the expected increase in STAI score based on the SCOPA-AUT cutoff. **(C)** Longitudinal trajectories of mean STAI changes in different SCOPA-AUT level groups.

Based on the linear mixed models and multivariable Cox model results, higher baseline SCOPA-AUT scores predicted the future anxiety progression and higher anxiety score during time. To further support the association between baseline SCOPA-AUT score and anxiety progression, SCOPA-AUT score cutoff values were defined as 18 based on the producing maximum log-rank score. Kaplan–Meier analyses showed that the “SCOPA-AUT ≥ 18” group (“high-level” group) had a significantly higher incidence of anxiety compared with the “SCOPA-AUT < 18” group (“low-level” group) (log-rank 12.8, *p* < 0.001) (Figure 2A), and the “high-level” group also had a faster rate of annual increase in STAI total score compared with the “low-level” group (Figures 2B,C).

**Figure 3:**
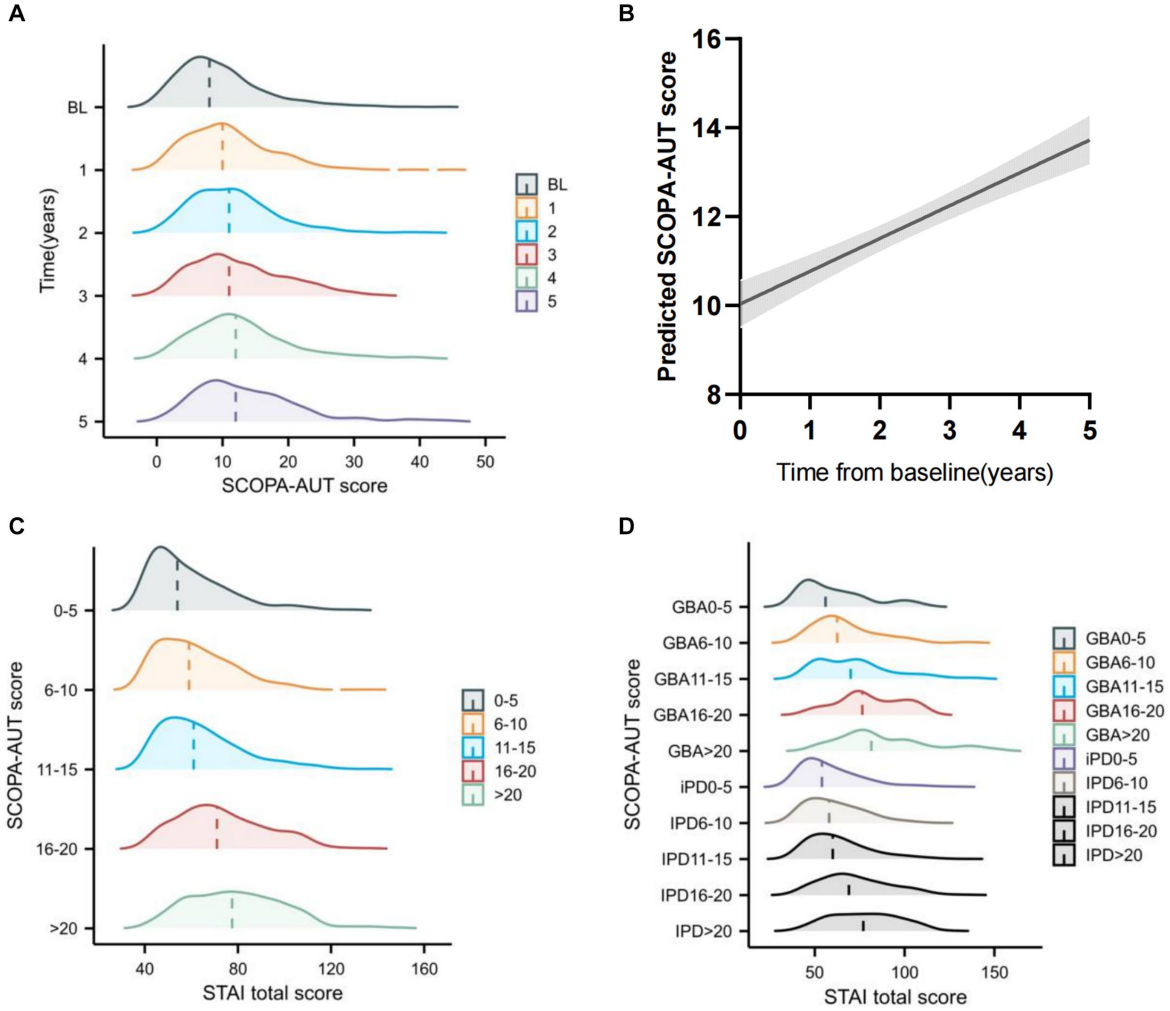
Longitudinal change of Scales for Outcomes in Parkinson’s Disease-Autonomic (SCOPA-AUT) and the association with State-Trait Anxiety Inventory (STAI) change over time **(A)** Raincloud plots showing the expected progressive increase in SCOPA-AUT score. **(B)** Longitudinal trajectories of SCOPA-AUT scores in patients with Parkinson’s disease. **(C)** Raincloud plots showing the expected STAI total score increase at different SCOPA-AUT score levels across all years. **(D)** Raincloud plots showing the expected STAI total score increase at GBA status and different SCOPA-AUT score levels across all years.

We also analyzed the SCOPA-AUT score over time and found that autonomic dysfunction increased over time (Figures 3A,B; Supplementary Figure S1). Dysautonomia was associated with higher anxiety score over time, and this trend was more significant in the *GBA* variant carriers (Figures 3C,D).

**Figure 4:**
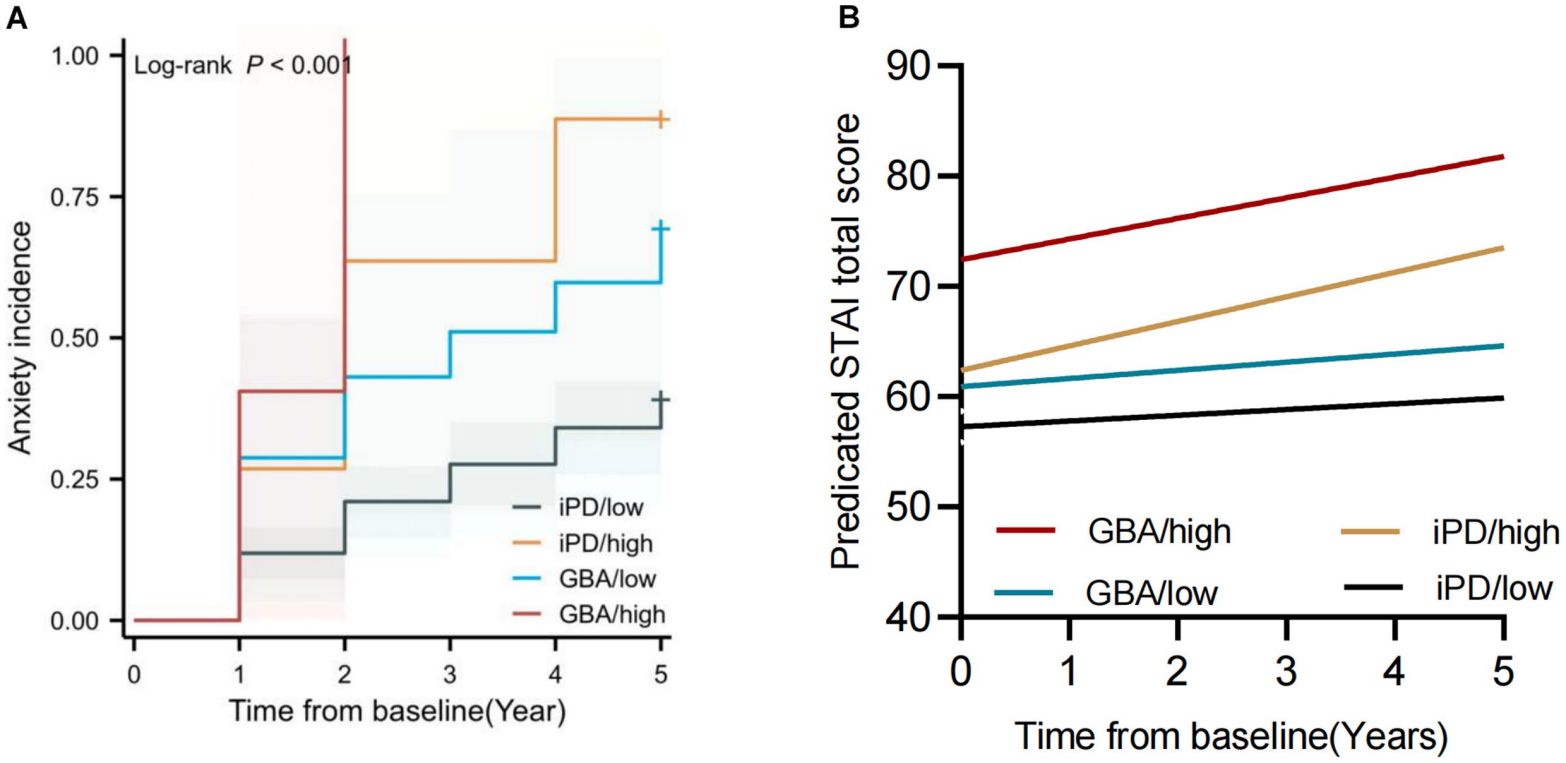
Longitudinal trajectories of the Mean State–Trait Anxiety Inventory (STAI) Rating **(A)** and Kaplan–Meier analyses of anxiety progression **(B)** in different groups according to the *GBA* variant carrier status and SCOPA-AUT level. “GBA/high” group: group with “*GBA* variants and SCOPA-AUT ≥ 18”; “GBA/low” group: group with “*GBA* variants and SCOPA-AUT < 18”; “iPD/high” group: group with “iPD and SCOPA-AUT ≥ 18”; “iPD/low” group: group with “iPD and SCOPA-AUT < 18”.

Next, we divided the patients into four groups based on the *GBA* variants and SCOPA-AUT level; the “GBA and SCOPA-AUT ≥ 18” group (“GBA/high” group) was at 7.5 times higher risk of progressing to anxiety than the “iPD and SCOPA-AUT < 18” group (“iPD/low” group), and the “iPD and SCOPA-AUT ≥ 18” group (“iPD/high” group) was at 2.3 times higher risk than the “iPD/low” group (log-rank 19.0, *p* = 0.004) (Figure 4A). Moreover, the anxiety score of the “GBA/high” and “iPD/high” group was predicted to increase much faster than the “GBA/low” and “iPD/low” groups (Figure 4B).

## Discussion

In this study, we first comprehensively explored the relationship between *GBA* variants and long-term anxiety progression in newly diagnosed patients, with PD followed from the time of diagnosis. Taking advantage of this longitudinal data from a well-designed cohort, our linear mixed model and multivariable Cox regression model showed that *GBA* variant carriers experienced more than 57% greater annual increase in STAI total anxiety score and faster progression in anxiety compared with non-carriers.

Previous studies assessing anxiety in GBA-associated PD were mostly cross-sectional studies, and *GBA* variant carriers presented with a high prevalence of anxiety (DeBroff et al., 2023). A few studies using univariable Kaplan–Meier survival analysis demonstrated that the GBA-PD group developed anxiety earlier than the idiopathic PD group (Pachi et al., 2021). In contrast with those studies, we observed a clear association between *GBA* status and increase in anxiety score over time, and this association remained significant after adding other clinical risk factors in the linear mixed model. In this adjusted linear mixed model, dysautonomia, depressive symptoms, and cognitive decline, which have been proposed in other studies, were also demonstrated to be predictors of increase in anxiety score (Rutten et al., 2017; Gibson et al., 2023).

Apart from *GBA* variants, dysautonomia was another independent anxiety predictor that ascertained both by linear mixed model and multivariable Cox regression model in this study. Autonomic dysfunction increased over time; dysautonomia was associated with higher anxiety score over time, and this trend was more significant in the *GBA* variant carriers. Since a consensus cutoff score is not available for dysautonomia based on SCOPA-AUT, we calculated a SCOPA-AUT cutoff score of 18 based on the log-rank survival analysis and the post-hoc analysis. The “high-level” group showed faster anxiety progression and increase in anxiety score over time. Moreover, the “GBA/high” group showed the fastest increase in anxiety score.

A previous study found that CSF t-tau and a-syn changes were associated with the increase in anxiety score over time (Wang et al., 2023). Our result found the CSF t-tau level increased over time, but the baseline CSF t-tau level was associated with the annual change in anxiety score. Recently, studies showed that tau protein accumulation showed age-dependent anxiety behaviors and exacerbate stress responses (Li et al., 2017; Liu et al., 2023), which, to some extent, support the previous finding and our results. It is well known that most *GBA* variants reduce GCase activity (Gegg et al., 2012), and GBA-PD may have a burden of iPD and demonstrated decreased CSF levels of total α-Syn compared with non-carriers in other cross-sectional studies (Lerche et al., 2021). However, in our study, we did not find the association between CSF a-syn burden and development of anxiety. But studies showed that tau protein could also accumulated in *GBA* mutation model (Yang et al., 2022b), and one drug that targeted the *GBA* variants carriers cohort ambroxol could reverses tau accumulation in *GBA* mutation model, which might suggest the tau protein’s role in the *GBA* variants’ contribution to the anxiety development in our results.

Unlike other studies, we did not find pRBD to be associated with anxiety symptoms over time, which might be associated with the *GBA* variant that we add in linear mixed model and Cox model for the first time. Studies have shown that *GBA* variant carriers developed pRBD more frequently than patients without *GBA* variants (Gan-Or et al., 2018; Huang et al., 2022); the pRBD that found by a previous cohort might be not an independent predictor but caused by the effect of *GBA* variants. This also encouraged us to explore the contribution of more genetic factors to the anxiety progression in PD.

Several limitations should be taken into consideration in this study. First, the retrospective design of this study represents its primary limitation. To mitigate this limitation, we attempted to address it by utilizing the linear mixed models and Cox survival analysis, to analyze the disease-duration scale time, employing log-rank tests for comparing survival curves. In addition, more prospective study on the prodromal *GBA* cohort, that did not develop PD yet, would help us to elucidate the contribution of *GBA* variants more clearly. Second, those non-motor symptoms including anxiety and dysautonomia were difficult to measure due to their unpredictable nature and excessive subjectivity. In future studies, these finding could be further supported with diagnostic confirmation of anxiety through neuroimaging and dysautonomia through polysomnography. Third, this PPMI cohort mainly consists of Caucasian population, and the Chinese population carries different *GBA* variants, so more studies need to replicate those results in the Chinese cohort to confirm our findings.

## Conclusion

Our finding provides evidence for the role of *GBA* variants in the rate of anxiety progression in the general PD population. We show that *GBA* variants and dysautonomia are independent predicators of faster increase in anxiety score and development. Considering this relationship, small molecules aimed at *GBA* variants and treatment for dysautonomia may help to reduce anxiety symptoms in people with PD. More studies need to be conducted to confirm our findings and reveal the precise mechanism, underlying the role of *GBA* variants in anxiety progression in the PD population and investigate the potential for anxiety reduction via the mechanism.

## Data availability statement

Publicly available datasets were analyzed in this study. This data can be found at: www.ppmi-info.org/access-ataspecimens/download-data.

## Ethics statement

The studies involving humans were approved by the ethics committee of First Affiliated Hospital of Zhengzhou University. The studies were conducted in accordance with the local legislation and institutional requirements. Written informed consent for participation was not required from the participants or the participants' legal guardians/next of kin in accordance with the national legislation and institutional requirements.

## Author contributions

SS: Data curation, Formal analysis, Funding acquisition, Investigation, Methodology, Writing – original draft. YB: Formal analysis, Writing – review & editing. NY: Conceptualization, Data curation, Formal analysis, Funding acquisition, Methodology, Project administration, Resources, Software, Validation, Visualization, Writing – original draft, Writing – review & editing.

## Footnotes

^1^ http://www.ppmi-info.org/study-design

^2^ http://ppmi-info.org/study-design
