# Supplementary material for: Longitudinal faster anxiety progression of GBA variant carriers in the early Parkinson’s disease cohort
Source: Front Neurosci. 2024 Jan 24;18:1353759. doi: 10.3389/fnins.2024.1353759 (PMC10847242; doi:10.3389/fnins.2024.1353759)
Supplement: Supplementary file 1 [file Table_1.docx]

| Characteristics variables | Mean (SD) |  | P value |
| --- | --- | --- | --- |
|  | iPD (n=346) | GBA-PD(n=39) |  |
| Age, y | 61.8(9.6) | 58.9(9.5) | 0.07 |
| Male, n (%) | 110(31.8) | 16(41.0) | 0.24 |
| Disease duration, y | 0.56(0.54) | 0.52(0.55) | 0.30 |
| Age at onset | 59.8 (9.7) | 56.9 (9.9) | 0.07 |
| STAI baseline | 56.2(10.4) | 58.5 (10.7) | 0.28 |
| MDS-UPDRS III | 20.4 (8.7) | 23.8(11.3) | 0.16 |
| PIGD subtype, n (%) | 101(29.2) | 11(28.2) | 0.89 |
| SCOPA-AUT | 9.7(6.8) | 10.3(7.1) | 0.71 |
| UPSIT score | 22.4 (8.2) | 21.7 (8.0) | 0.49 |
| MoCA score | 27.0 (2.4) | 26.7 (2.4) | 0.25 |
| RBDSQ score | 3.9 (2.6) | 5.8 (3.0) | **< 0.001** |
| pRBD(RBDSQ>5) | 84(24.2) | 18(46.2) | **0.003** |
| GDS score | 2.4 (2.6) | 2.7 (2.8) | 0.57 |
| Depression (GDS>5) | 40(11.6) | 6(15.3) | 0.66 |
| ESS score | 5.9 (3.5) | 6.1 (3.7) | 0.76 |
| EDS (ESS>10) | 55(15.9) | 8(20.5) | 0.46 |
| Caudata DAT | 2.0 (0.58) | 1.9 (0.64) | 0.14 |
| Putamen DAT | 0.88(0.41) | 0.76 (0.30) | 0.06 |
| CSF Aβ_42_ | 889.3(421.8) | 807.9 (341.6) | 0.48 |
| CSF a-syn | 1473.1 (691.1) | 1315.2 (621.4) | 0.25 |
| CSF t-tau | 161.1 (66.8) | 144.4 (65.2) | 0.32 |
| CSF p-tau | 13.3(5.8) | 12.0(5.9) | 0.39 |

**Table 1 Characteristics of the *GBA* variants carriers and iPD**

Data are shown as mean ± standard deviation or prevalence(%); Abbreviations: GBA= β-glucocerebrosidase; STAI=State-Trait Anxiety Inventory; MDS-UPDRS III =Movement Disorders Society Unified Parkinson’s Disease Rating Scale part3; PIGD = postural instability gait difficulty; SCOPA=Scale for Outcomes in Parkinson disease Autonomic; RBDSQ=REM Sleep Behavior Disorder Screening Questionnaire; UPSTI= University of Pennsylvania Smell Identification Test; GDS=Geriatric Depression Scale; MoCA=Montreal Cognitive Assessment; ESS=Epworth sleepiness Scale; EDS=Excessive Daytime Sleepiness; DAT = dopamine transporter; CSF= cerebrospinal fluid; Aβ42= β-amyloid 1-42; α-syn=total alpha-synuclein; t-tau=total tau; p-tau= phosphorylated tau. Values in bold indicate statistically significant results.
